# Problem Gambling Transmission. An Eight-wave Longitudinal Study on Problem Gambling Among Affected Others

**DOI:** 10.1007/s10899-025-10465-0

**Published:** 2025-12-22

**Authors:** Emmi Kauppila, Sari Hautamäki, Iina Savolainen, Sari Castrén, Richard Velleman, Atte Oksanen

**Affiliations:** 1 Faculty of Social Sciences, Tampere University, Tampere, Finland; 2 A-Clinic Foundation, Helsinki, Finland; 3 Finnish Institute for Health and Welfare, Health and Well-Being Promotion Unit, Helsinki, Finland; 4 Social Sciences, Department of Psychology and Speech-Language Pathology, University of Turku, Turku, Finland; 5 Department of Medicine, University of Helsinki, Helsinki, Finland; 6 Department of Psychology, University of Bath, Bath, UK; 7 Addictions and Related Research Group, Sangath Community Health NGO, Goa, India

**Keywords:** Problem gambling, Affected others, Concerned significant others, Social connectedness

## Abstract

There is growing recognition that those close to someone with problem gambling experience stress and strain. Research suggests they may also be at risk of developing gambling problems themselves, but this remains an underexplored area. The present study examined how exposure to problem gambling within family or friend networks relates to affected others’ own gambling, and whether strong social connections reduce this risk. Using an eight-wave longitudinal dataset (*N* = 1530) with hybrid multilevel regression modeling, we analyzed within-person and between-person effects of exposure to a family member’s or friend’s problem gambling on affected others’ own gambling. We further examined the protective role of social connectedness to family and friends. Gambling problems were assessed with the Problem Gambling Severity Index (PGSI) and social relationships with the Social and Emotional Loneliness Scale for Adults (SELSA). Results indicated that individuals were more likely to develop problem gambling when they reported that a family member or a friend had gambling problems. Strong family relationships buffered against this risk, whereas friendships did not provide the same protection. These findings suggest that social relationships play an important role in shaping problem gambling among affected others and highlight the need to consider family and peer contexts in prevention and intervention strategies.

## Introduction

In recent years, research on gambling harm has expanded beyond the individual gambler to include harms experienced by families, communities, and broader social networks (Hautamäki et al., 2025; Langham et al., 2015). Affected others, such as family members, close friends, and partners, are increasingly recognized as bearing significant emotional, relational, financial, and health-related burdens (Dowling et al., 2025a; Merkouris et al., 2022; Riley et al., 2021; Tulloch et al., 2022).

In Finland, approximately 20% of adults (around 722,000 individuals) identify as affected others of someone else’s gambling (Grönroos et al., 2024). A recent scoping review estimates the prevalence of affected others in the general population to range from 4.5 to 21.2% (Dowling et al., 2025b). Emotional harm is the most commonly reported negative consequence, followed by relationship, financial, and health harms (Riley et al., 2021). Women, particularly former or current partners, are more likely to experience multiple and severe harms, including psychological distress and poor health (Hing et al., 2022; Lind et al., 2022). Affected family members report higher harm exposure than affected close friends, and are more likely to face financial hardship, risky alcohol use, and co-occurring problem gambling issues (Lind et al., 2022). Additionally, children in gambling-affected families may experience severe consequences, including psychological distress (e.g., anxiety, depression, suicidality), family violence, and neglect, underscoring the need for public health responses (Suomi et al., 2022).

One consequence of being close to someone with problem gambling is an increased risk of developing similar issues, a pattern known as problem gambling transmission (Dowling et al., 2017, 2018, 2021; Kourgiantakis et al., 2016). Studies in this area often focus on intergenerational transmission of problem gambling, with longitudinal studies providing evidence of the association between parental gambling exposure and an individual’s likelihood of developing gambling problems later in life (Forrest & McHale, 2021; Vitaro et al., 2008; Winters et al., 2002). Similar intergenerational patterns have long been documented in alcohol research, where parental substance use is understood to affect children not only through direct exposure, but also via broader familial dynamics such as conflict, neglect, or emotional distance (Velleman, 1992a, 1992b).

Evidence also suggests that exposure to problem gambling in other relationships may increase individual’s risk of developing similar issues (Lind et al., 2022; Salonen et al., 2014; Wilson et al., 2024). Being friends with a person with problem gambling is associated with higher levels of gambling participation and risk (Castrén et al., 2021). Similarly, peer influence and shared gambling experiences among friends and colleagues can lead to increased gambling frequency (Newall et al., 2024). As Reith and Dobbie (Reith & Dobbie, 2011) suggest, gambling may be better understood not only as an inherent trait but also as a behavior shaped and reinforced through social interaction.

Although perceived peer gambling consistently predicts problem gambling in adolescence (Parrado-González et al., 2023; Savolainen et al., 2019; Zhai et al., 2017), gambling behavior transmission in adult friendships has been studied less often. Existing studies have relied mainly on cross-sectional or qualitative designs (Castrén et al., 2021; Deans et al., 2017; Lind et al., 2022), and longitudinal evidence on how peer gambling influences adult gambling behavior over time is still lacking.

On the other hand, family and friend relationships can also offer a protective factor against problem gambling (Smith et al., 2025). Family connectedness has been identified as one of the key protective factors against gambling problems, associated with reduced gambling problems in both adolescence and adulthood. In particular, parental monitoring and positive parent–child relationships have been shown to significantly lower the risk of youth gambling, with evidence indicating that active oversight reduces gambling involvement (Castrén et al., 2021), while strong emotional bonds and engagement in meaningful activities further buffer against gambling-related harms (Pisarska & Ostaszewski, 2020). Friend relationships, while offering a delayed and less consistent protective effect, may still contribute to lower risk in early adulthood (Allami et al., 2021). The type of friendship may matter: offline belonging can protect against gambling problems, while online belonging may increase risk among young people (Savolainen et al., 2019).

While prior research highlights both risk and protective roles of family and peer relationships, the evidence base is heavily weighted toward adolescent populations and intergenerational transmission. Far less is known about how these dynamics operate in adulthood. Understanding these mechanisms is essential for developing prevention and intervention strategies that leverage social relationships as protective resources. Building on this existing knowledge, this study aims to clarify the distinct roles that family and friend relationships play in influencing gambling transmission among adults. This study addresses the following research questions:

- RQ1: Does exposure to problem gambling within family and friends increase the risk of developing problem gambling over time?
- RQ2: Do strong family and friend relationships buffer the impact of such exposure?

## Methods

### Participants and Procedure

The eight-wave longitudinal data used in this study were collected within the [anonymized for review] research project between April 2021 and September 2024. A survey design was used, and data collections were conducted at six-month intervals. The survey was designed by the research team in collaboration with a data provider company Norstat Finland. Participants were recruited from Norstat Finland’s online panel of adults aged 18–75 residing in mainland Finland. Norstat invited panel members via email based on predefined demographic quotas for age, gender, and region to ensure the sample reflected the mainland Finnish adult population. A comparison with Statistics Finland census data showed that the sample closely matched the national population distribution in terms of age and gender, with only minor deviations in regional distribution and educational attainment (Oksanen et al., 2022)anonymized for review). Based on this demonstrated representativeness, unweighted data were used.

In the first data collection point, the response rate was 34.60% (*N* = 1530). All T1 respondents were re-invited to the follow-up surveys. Over the eight waves, the sample consistently maintained demographic representativeness, both internally and in comparison with the Finnish mainland adult population. The response rate has remained high in the follow-up surveys: T2 (*n* = 1198, response rate 78% of T1 respondents), T3 (*n* = 1095, response rate 72% of T1 respondents), T4 (*n* = 1004, response rate 66% of T1 respondents), T5 (*n* = 934, response rate 61% of T1 respondents), T6 (*n* = 889, response rate 58% of T1 respondents) T7 (*n* = 873, response rate 57% of T1 respondents) and T8 (*n* = 824, response rate 54% of T1 respondents). The participants spent around 15 min on each survey. At T1, the sample included adults aged 18–75 years (*M* = 46.7, *SD* = 16.4), with an even gender distribution (50% male) and educational attainment comparable to the Finnish adult population (39% with a bachelor’s degree). Income levels averaged 3.1 (*SD* = 1.6) on an eight-point scale, indicating a modal income of 2000–2999 euros. Demographic composition remained stable across waves, supporting the representativeness of the panel (see Table 1 for full descriptive statistics).

**Table 1:** Descriptive statistics of the data at the eight timepoints

Categorical variable		T1	T2	T3	T4	T5	T6	T7	T8
		**%** **(*n*)**	**%** **(*n*)**	**%** **(*n*)**	**%** **(*n*)**	**%** **(*n*)**	**%** **(*n*)**	**%** **(*n*)**	**%** **(*n*)**
AO: Family member with GP		11.8 (181)	5.3 (64)	5.8 (64)	6.2 (62)	5.7 (53)	5.9 (52)	5.8 (51)	5.5 (45)
AO: Friend with GP		10.1 (155)	7.2 (86)	5.8 (63)	6.3 (63)	5.8 (54)	5.9 (52)	5.0 (44)	5.0 (41)
Gender (male)		50.3 (770)	50.7 (607)	50.1 (549)	50.4 (506)	50.5 (472)	50.2 (446)	50.4 (440)	50.6 (417)
Bachelor’s degree		38.5 (589)	39.9 (478)	39.4 (432)	41.5 (417)	41.2 (385)	42.3 (376)	41.6 (363)	41.6 (343)
Continuous variable	Range	***M*** (***SD***)	***M*** (***SD***)	***M*** (***SD***)	***M*** (***SD***)	***M*** (***SD***)	***M*** (***SD***)	***M*** (***SD***)	***M*** (***SD***)
GP	0–25	1.3 (3.3)	1.2 (3.2)	1.2 (3.2)	1.1 (2.9)	1.0 (2.8)	0.9 (2.6)	0.9 (2.7)	0.9 (2.9)
Close relationship with family	3–21	17.8 (3.9)	17.8 (4.0)	17.5 (4.0)	17.7 (4.0)	17.7 (3.8)	17.7 (4.0)	17.9 (4.0)	17.7 (4.2)
Close relationship with friends	3–21	16.2 (4.5)	15.9 (4.7)	15.7 (4.6)	15.9 (4.7)	16.0 (4.5)	15.8 (4.7)	15.9 (4.9)	16.1 (4.6)
Age (T1)	18–75	46.7 (16.4)	48.4 (16.1)	48.7 (16.2)	49.2 (15.9)	49.9 (15.4)	50.6 (15.3)	50.5 (15.3)	50.7 (15.4)
Monthly income	1–8	3.1 (1.6)	3.1 (1.6)	3.2 (1.6)	3.3 (1.6)	3.4 (1.6)	3.4 (1.6)	3.4 (1.7)	3.5 (1.6)
Total, *N*		1530	1198	1095	1004	934	889	873	824

*AO* affected other, *GP* gambling problems (pgsi), *M* mean, *SD* standard deviation, *T1* timepoint 1

Steps to ensure quality of the data were taken by both the data provider company and the research team. Norstat regularly compares the panel member profiles with official population statistics and employs targeted participant recruitment to address gaps in demographics. For this study, demographic quotas were applied during baseline wave (T1), and subsequent waves consisted of recontacting T1 respondents without additional top-up sampling. Following a predefined protocol, the research team performed quality checks to identify and exclude participants displaying consistent or logical biases in their responses. This also included assessment of completion times to identify unusually rapid submissions. All cases flagged as invalid were removed from the dataset before conducting analyses.

### Measures

Affected other status was determined by asking respondents whether they had a close relationship with someone who had experienced gambling problems. They could select multiple relationship categories, including 'Mother,' ‘Father’, ‘Child’, 'Sibling,' 'Partner,' 'Grandparent', or 'Friend’. At T1, the question referred to any point in the respondent’s life to establish a broader context of perceived problem gambling in close relationships before narrowing the timeframe to six months in later waves to track changes over time. From wave 2 onward, the question was modified to cover only the past six months to align with the longitudinal design. This change in timeframe likely explains the higher proportion of affected others at T1 compared to subsequent waves.

Based on these responses, two binary variables were created: one indicating whether the respondent reported gambling problems of a family member (parent, sibling, partner, child, or grandparent), and another indicating whether a close friend was affected. Respondents could be marked as having both types of affected relationships. Although confirming whether the person of concern meets problem gambling criteria falls outside the scope of this study, research shows that the mere belief that there is addiction within the family—even if these perceptions are not accurate—is linked to increased severity in gambling problems (Felsher et al., 2003). Although consequences may differ across relationship roles, separate role-specific analyses were not conducted because the number of affected others in each subgroup (e.g., partner, parent, grandparent) was insufficient for reliable estimation in this study.

Problem gambling was assessed with the widely used general population screener for excessive gambling: the Problem Gambling Severity Index (Ferris & Wynne, 2001). The scale comprises nine items assessing different dimensions and consequences of gambling behavior. Respondents rate each item on a 0 to 3 scale (0 = Never, 1 = Sometimes, 2 = Most of the time, 3 = Almost always), yielding an overall score from 0 to 27. Higher scores indicate a greater likelihood of problem gambling. While the original measure examines gambling over the past 12 months, the timeframe was adjusted to the last 6 months to correspond with the data collection period. Previous research has shown that shortening the timeframe of gambling severity instruments maintains reliability and validity (Wulfert et al., 2005).

Social connectedness was evaluated using the Social and Emotional Loneliness Scale for Adults (SELSA; DiTommaso & Spinner, 1993). The scale features three subscales addressing family, friends, and a loved one, each containing three statements (for example, ‘I can depend upon my friends for help’; My family is important to me’). In this analysis, we used the sub-scales for family and friends, each containing three items. Respondents rated their agreement on a 7-point scale ranging from 1 (strongly disagree) to 7 (strongly agree), with scores ranging from 3 to 21 for each scale. The SELSA measures were standardized and re-scaled to have a mean of zero to ensure even distribution between timepoints. Age and gender at the first timepoint, education level (classified as at least a bachelor's degree vs. lower), and monthly income (ranging from 1 = less than €1,000 to 8 = €7,000 or more) were used as control variables. Descriptive statistics of these variables across the eight timepoints are presented in Table 1.

### Statistical Analysis

For the main analyses, hybrid multilevel linear regression models were employed using the xthybrid command on Stata version 17 statistical software (Stata Corp). By combining the strengths of fixed- and random-effects models, hybrid models are well-suited for examining the within- and between-person associations in longitudinal studies (Schunck, 2013; Schunck & Perales, 2017). The fixed effects reveal within-individual associations between time points for time variant variables. This enables examination of possible changes over time in the relationship between dependent and independent variables for each individual. The random effects examination allows to control time-invariant variables in the model, also giving the values based on between individual comparison (Schunck & Perales, 2017). No item-level missingness occurred because the survey format did not allow respondents to skip questions. Missing data arose solely from participants not responding in all waves. Hybrid models incorporate each participant’s available observations across waves, allowing respondents with partial follow-up to be retained in the analysis and helping to reduce loss of power and bias related to missing data.

Problem gambling, measured by the PGSI score, was the dependent variable. The independent variables included problem gambling within the family, problem gambling by a friend, and sense of connectedness to family and friends (measured by the SELSA scores). In addition, moderation was examined including interaction terms between the affected other variables and the social relationship measures in separate fixed effects regression models. Additionally, we run sensitivity analyses using categorical variables for PGSI. These were conducted with outcome variables indicating both moderate (PGSI ≥ 3) and severe (PGSI ≥ 8) risk for problem gambling. The results were consistent with our main results.

## Results

The results of the hybrid multilevel model showed that having a family member with problem gambling had a statistically signficant within-person effect on problem gambling (*B* = 0.61, *p* =.006, see Table 2). This means that the PGSI score increases by 0.61 units for individuals who newly report that their family member has a gambling problem. Having a friend with problem gambling also seemed to increase the risk of problem gambling, however, did not reach statistical significance (*B* = 0.27, *p* =.052) and the effect was small in magnitude.

**Table 2:** Within and between-person estimates from hybrid regression models

	B	SE (B)	p
Within-person			
Family member with GP	0.61	0.22	.006
Friend with GP	0.27	0.14	.052
Relationship with family	−0.02	0.01	.059
Relationship with friends	−0.02	0.01	.059
Income	0.03	0.03	.396
Between-person			
Age	−0.02	0.00	<.001
Male	0.66	0.14	<.001
BA degree or higher	−0.34	0.14	.016
Family member with GP	3.65	0.56	<.001
Friend with GP	2.17	0.43	<.001
Relationship with family	−0.07	0.02	.001
Relationship with friends	−0.05	0.02	.015
Income	0.04	0.06	.50

Between individuals, both variables strongly associated with problem gambling, indicating that those with family members (*B* = 3.65, *p* <.001) and a friend (*B* = 2.17, *p* <.001) with problem gambling were more likely to experience problem gambling themselves. It is notable that these between-person effects are large in size. For example, participants who have family members with a gambling problem report a 3.65 point higher PGSI score compared to those who do not have affected family members. An increase of 3.65 in the PGSI could be considered substantial. Similarly, a 2.17 higher score on the PGSI among those who had a friend with gambling problem could be considered high. We also found between-person effects in close relationships with family and friends. The results showed that having a close relationship with family (β = −0.07, *p* = 0.001) or friends (β = −0.05, *p* = 0.015) was associated with a lower risk of experiencing problem gambling.

**Fig. 1:**
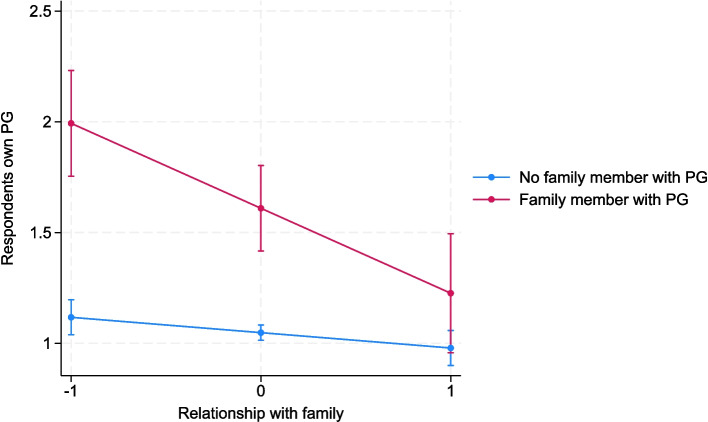
Interaction effects of family relationships on respondents’ own problem gambling within individual

To examine the association of close family relationships within individuals, an interaction analysis was conducted. It revealed that having a close relationship with family moderated the association between having someone close in the family with gambling problems and respondents own gambling problems within individuals (see Fig. 1). Thus, according to these results, having close relationships within family acts as a buffer against problem gambling transmission. However, having a close relationship with friends was not moderating the association between having a friend with problem gambling and the respondents’ own problem gambling.

## Discussion

To advance understanding of factors influencing gambling behavior, research increasingly highlights the role of family and friends (Allami et al., 2021; Ghelfi et al., 2023). The findings of this study show that exposure to problem gambling among both family members and friends predicted increases in individuals own problem gambling over time. Fulfilling family relationships buffered this effect, whereas strong relationships with friends did not show a comparable buffering effect.

The finding that exposure to problem gambling predicts increases in one’s own problem gambling is consistent with long-standing evidence on familial transmission of gambling behavior. Prior work has shown that exposure to parental or sibling gambling, and the modeling, normalization and shared family norms that accompany it, heighten the likelihood of gambling problems in adulthood (Canale et al., 2017; Dowling et al., 2018; Suomi et al., 2022) (Dowling et al., 2021; Suomi et al., 2022). Although the current study could not directly examine these mechanisms, its results align with this broader evidence and extend it by showing that social exposure remains influential in a general adult population, beyond youth or clinical samples.

The finding that fulfilling family relationships buffered the effect of exposure to problem gambling extends a limited body of work on protective family processes. Prior research has shown that positive parenting practices, responsive communication, and emotionally supportive family environments can mitigate the intergenerational transmission of gambling-related harm (Suomi et al., 2022). The present results contribute to the evidence by showing that supportive family relationships also reduce risk among adults, suggesting that exposure effects are not uniform but shaped by the quality of family interactions. This highlights the importance of considering relationship quality, rather than exposure alone, when assessing gambling-related vulnerabilities within families.

The finding that strong friendships did not protect against the transmission of gambling harm may be understood in light of social withdrawal. Across alcohol and substance use studies, affected others have been found to isolate from friends or conceal their situation due to shame or the belief that others will not understand (Velleman et al., 2025). Research on alcohol and substance use points out that stigma, fear of blame, and cultural expectations on privacy can discourage open discussion (Lindeman & Selseng, 2025). These patterns may help explain why even close peer relationships, while emotionally supportive, do not interrupt the transmission of gambling harm. Another way to interpret this finding draws on broader addiction literature, which highlights how people may be drawn to relationships that reinforce, rather than buffer, patterns of harm. An early study by Plant (n.d.) investigated whether working in the alcohol industry increased the risk of harmful drinking. He found evidence for both self-selection and environmental reinforcement: individuals with heavier drinking patterns were more likely to enter the industry, and the work environment itself tended to support continued or escalating use. A similar pattern may help explain the findings: Rather than disrupting harmful dynamics, some peer relationships may reflect a pre-existing alignment with gambling behaviors or shared coping strategies that maintain rather than mitigate harm. This interpretation finds support in gambling research that identifies workplace dynamics, such as social norms and environmental cues, as contributing to the persistence of harmful behaviors (Hing & Gainsbury, 2013).

The findings of this study add to research showing that when gambling is seen as normal by others, it can increase a person’s own risk of gambling (León-Jariego et al., 2020; Meisel & Goodie, 2014; Parrado-González et al., 2023). A recent review found that early exposure to gambling within family settings was common among adults affected by gambling harm (Smith et al., 2025). Moreover, recent studies have reinforced that gambling harm tends to cluster within social networks: individuals engaged in high-risk gambling are often embedded in environments where others also experience gambling-related harms (Savolainen et al., 2019; Vepsäläinen et al., 2024). A contributing factor may be that witnessing or participating in gambling within close relationships, especially among peers or family, reinforces the normalization of these behaviors. This interpretation resonates with findings from several studies showing that individuals are more likely to gamble when they perceive that close friends approve of gambling or expect them to participate (Dahl et al., 2018; Deans et al., 2017; Gay et al., 2016; Martin et al., 2010). These insights underpin emerging prevention strategies such as the PRoGRAM-A intervention, which uses peer-led social network approaches in school settings to disrupt the social reinforcement of gambling among adolescents (Dobbie et al., 2024). This approach, for example, underscores the importance of considering social contexts in the development and implementation of effective gambling harm prevention strategies.

As hybrid models distinguish within-person and between-person associations (Schunck, 2013; Schunck & Perales, 2017), the findings can be interpreted at two levels. The within-person estimates show how changes in exposure to problem gambling within an individual’s own life corresponded with changes in their gambling behavior across waves. The between-person estimates capture more stable differences across respondents, indicating that individuals with family members or friends who gamble problematically tend to report higher levels of gambling problems overall. Together, these components suggest that both situational changes and more enduring social environments contribute to problem gambling transmission.

### Clinical Implications

Mounting evidence highlights the critical importance of addressing the needs of affected family members in gambling harm prevention and treatment. A systematic review and meta-analysis by Vassallo et al. (2023) found that while many interventions show promise, their overall effectiveness remains limited. Both this study and earlier work (Rodda et al., 2020) underscore the necessity of designing programs that directly prioritize the wellbeing of affected others rather than focusing solely on the gambler’s recovery. Similarly, Smith et al. (2025) emphasize that families shape gambling behaviors through early normalization of gambling, strained parental relationships, and exposure to conflict, which can increase the risk of gambling harm across the life course. Despite this, they argue that family dynamics remain underexamined in both research and practice, calling for greater inclusion of families in harm reduction strategies. In support of this, Tremblay et al. (2015) present an integrative couple treatment model, which illustrates how working with both the gambler and their partner can improve psychological wellbeing, enhance relationship satisfaction, and facilitate gambling cessation. Including family members in therapy not only benefits the gambler but also addresses the distress and relational strain experienced by those closest to them.

Recognizing the experiences of affected family members is critical in responding effectively to gambling harm. Affected family members are not only passive witnesses to addiction but often experience severe and long-lasting harm themselves, which may manifest as anxiety, depression, family conflict, and social isolation (Bischof et al., 2025). There is increasing recognition that a systemic approach including affected family members in both assessment and intervention can mitigate harm and promote resilience. Providing appropriate psychosocial services, improving access to information, and recognizing the unique needs of different family members can reduce the intergenerational impact of gambling and foster recovery environments. This perspective underlines the urgent need to embed family-focused strategies in gambling harm prevention and treatment systems to ensure a more comprehensive response.

### Strengths and Limitations

The scope of this study was limited to Finnish context. In Finland, gambling is widely accepted, both through historical integration with the welfare state and through the continued public perception of gambling as a normal and even beneficial activity (Matilainen, 2016; Matilainen & Raento, 2014). By contrast, in countries where gambling is heavily restricted —such as Norway, where gambling is also state-controlled but access is more limited—gambling behaviors and related harms may manifest differently, highlighting the need for comparative cross-cultural research. Another limitation of the study is that the analysis did not identify which family member had the gambling problem. This was due to the small number of cases in certain categories, the limited variation in problem gambling severity, and differences in the strength and quality of the family relationship. These could be important examination points for future research. Moreover, a limitation of this study was that affected other status was defined by exposure to problem gambling rather than the experience of gambling harm. This approach may underestimate harm occurring in relationships with individuals who gamble at lower risk levels, as research shows that harm can occur across the continuum of gambling involvement (Dowling et al., 2025b). A key strength of our study is the longitudinal and nationally representative dataset, providing much-needed longitudinal evidence on affected others and focusing on adults – majority of research in this area has relied on cross-sectional and qualitative designs (Dowling et al., 2025b).

## Conclusions

This study set out to explore how exposure to problem gambling in family or friends relates to affected others’ own risk of developing similar issues, and to examine the role of social connectedness in buffering this risk. Results show that exposure within either network increases risk, but strong family ties—not friendships—provide a buffering effect. By highlighting the distinct roles of family and peer relationships, this study informs future intervention and prevention efforts aimed at mitigating problem gambling risk in affected others.

## Data Availability

The data that support the findings of this study are available on request from Author 6.

## References

[CR1] Allami, Y., Hodgins, D. C., Young, M., Brunelle, N., Currie, S., Dufour, M., Flores-Pajot, M., & Nadeau, L. (2021). A meta-analysis of problem gambling risk factors in the general adult population. *Addiction,**116*(11), 2968–2977. 10.1111/add.15449 33620735 10.1111/add.15449 PMC8518930

[CR2] Bischof, G., Velleman, R., Orford, J., Nadkarni, A., & Tiburcio, M. (Eds.). (2025). *Families affected by addiction: A handbook*. Springer Nature Switzerland. 10.1007/978-3-031-82583-5

[CR3] Canale, N., Vieno, A., Griffiths, M. D., Siciliano, V., Cutilli, A., & Molinaro, S. (2017). “I am becoming more and more like my eldest brother!”: The relationship between older siblings, adolescent gambling severity, and the attenuating role of parents in a large-scale nationally representative survey study. *Journal of Gambling Studies,**33*(2), 425–435. 10.1007/s10899-016-9643-5 27718036 10.1007/s10899-016-9643-5

[CR4] Castrén, S., Lind, K., Hagfors, H., & Salonen, A. H. (2021). Gambling-related harms for affected others: A Finnish population-based survey. *International Journal of Environmental Research and Public Health,**18*(18), 9564. 10.3390/ijerph18189564 34574487 10.3390/ijerph18189564 PMC8465844

[CR5] Dahl, E., Tagler, M. J., & Hohman, Z. P. (2018). Gambling and the reasoned action model: Predicting past behavior, intentions, and future behavior. *Journal of Gambling Studies,**34*(1), 101–118. 10.1007/s10899-017-9702-6 28623608 10.1007/s10899-017-9702-6

[CR6] Deans, E. G., Thomas, S. L., Daube, M., & Derevensky, J. (2017). The role of peer influences on the normalisation of sports wagering: A qualitative study of Australian men. *Addiction Research & Theory,**25*(2), 103–113. 10.1080/16066359.2016.1205042

[CR58] DiTommaso, E., & Spinner, B. (1993). The development and initial validation of the Social and Emotional Loneliness Scale for Adults (SELSA). *Personality and Individual Differences,**14*(1), 127–134. 10.1016/0191-8869(93)90182-3

[CR7] Dobbie, F., Miller, M., Wardle, H., Dahlby, L., Weir, C., Niven, A., Stoddart, A., Griffiths, D., Lee, A., Good, S., Noble, L., & White, J. (2024). Protocol for a pilot cluster randomised controlled trial of PRoGRAM-A (preventing gambling-related harm in adolescents): A secondary school-based social network intervention. *Pilot and Feasibility Studies,**10*(1), 109. 10.1186/s40814-024-01537-w 39138530 10.1186/s40814-024-01537-w PMC11321010

[CR8] Dowling, N. A., Shandley, K. A., Oldenhof, E., Affleck, J. M., Youssef, G. J., Frydenberg, E., Thomas, S. A., & Jackson, A. C. (2017). The intergenerational transmission of at-risk/problem gambling: The moderating role of parenting practices. *American Journal on Addictions,**26*(7), 707–712. 10.1111/ajad.12599 28881065 10.1111/ajad.12599

[CR9] Dowling, N. A., Oldenhof, E., Shandley, K., Youssef, G. J., Vasiliadis, S., Thomas, S. A., Frydenberg, E., & Jackson, A. C. (2018). The intergenerational transmission of problem gambling: The mediating role of offspring gambling expectancies and motives. *Addictive Behaviors,**77*, 16–20. 10.1016/j.addbeh.2017.09.003 28941932 10.1016/j.addbeh.2017.09.003

[CR10] Dowling, N. A., Francis, K. L., Dixon, R., Merkouris, S. S., Thomas, S. A., Frydenberg, E., & Jackson, A. C. (2021). “It runs in your blood”: Reflections from treatment seeking gamblers on their family history of gambling. *Journal of Gambling Studies,**37*(2), 689–710. 10.1007/s10899-020-09959-w 32671673 10.1007/s10899-020-09959-w

[CR11] Dowling, N. A., Hawker, C. O., Merkouris, S. S., Rodda, S. N., & Hodgins, D. C. (2025a). Addressing gambling harm to affected others: A scoping review (part I: Prevalence, socio-demographic profiles, gambling profiles, and harm). *Clinical Psychology Review,**116*, 102542. 10.1016/j.cpr.2025.102542 39914105 10.1016/j.cpr.2025.102542

[CR12] Dowling, N. A., Hawker, C. O., Merkouris, S. S., Rodda, S. N., & Hodgins, D. C. (2025b). Addressing gambling harm to affected others: A scoping review (part II: Coping, assessment and treatment). *Clinical Psychology Review,**116*, 102543. 10.1016/j.cpr.2025.102543 39854974 10.1016/j.cpr.2025.102543

[CR13] Felsher, J. R., Derevensky, J. L., & Gupta, R. (2003). Parental influences and social modelling of youth lottery participation. *Journal of Community & Applied Social Psychology,**13*(5), 361–377. 10.1002/casp.738

[CR14] Ferris, J., & Wynne, H. (2001). *The Canadian problem gambling index*. Canadian Centre on Substance Abuse.

[CR15] Forrest, D., & McHale, I. G. (2021). Transmission of problem gambling between adjacent generations. *Journal of Gambling Studies,**37*(2), 711–722. 10.1007/s10899-020-09977-8 32960384 10.1007/s10899-020-09977-8 PMC8144084

[CR16] Gay, J., Gill, P. R., & Corboy, D. (2016). Parental and peer influences on emerging adult problem gambling: Does exposure to problem gambling reduce stigmatizing perceptions and increase vulnerability? *Journal of Gambling Issues,**33*, 30. 10.4309/jgi.2016.33.3

[CR17] Ghelfi, M., Scattola, P., Giudici, G., & Velasco, V. (2023). Online gambling: A systematic review of risk and protective factors in the adult population. *Journal of Gambling Studies,**40*(2), 673–699. 10.1007/s10899-023-10258-3 37964161 10.1007/s10899-023-10258-3 PMC11272810

[CR18] Grönroos, T., Salonen, A., Latvala, T., Kontto, J., & Hagfors, H. (2024). Finnish gambling 2023. Gambling has decreased, gambling problems have become more prevalent and attitudes towards gambling have changed (Statistical Report No. 15/2024; Statistical Reports of the Finnish Institute for Health and Welfare). Finnish Institute for Health and Welfare (THL). https://www.julkari.fi/bitstream/handle/10024/148897/10.11.%20Statistical_report_15_2024.pdf?sequence=9&isAllowed=y.

[CR19] Hautamäki, S., Marionneau, V., Castrén, S., Palomäki, J., Raisamo, S., Lintonen, T., Pörtfors, P., & Latvala, T. (2025). Methodologies and estimates of social costs of gambling: A scoping review. *Social Science & Medicine,**371*, 117940. 10.1016/j.socscimed.2025.117940 40081164 10.1016/j.socscimed.2025.117940

[CR20] Hing, N., & Gainsbury, S. (2013). Workplace risk and protective factors for gambling problems among gambling industry employees. *Journal of Business Research,**66*(9), 1667–1673. 10.1016/j.jbusres.2012.12.013

[CR21] Hing, N., Browne, M., Rockloff, M., Tulloch, C., Rawat, V., Greer, N., Dowling, N. A., Merkouris, S. S., King, D., Stevens, L., Salonen, A. H., Breen, H., & Woo, L. (2022). Gambling-related harms to concerned significant others: A national Australian prevalence study. *Journal of Behavioral Addictions,**11*(2), 361–372. 10.1556/2006.2022.00045 35895474 10.1556/2006.2022.00045 PMC9295213

[CR22] Kourgiantakis, T., Stark, S., Lobo, D. S. S., & Tepperman, L. (2016). Parent problem gambling: A systematic review of prevention programs for children. *Journal of Gambling Issues,**33*, 8. 10.4309/jgi.2016.33.2

[CR23] Langham, E., Thorne, H., Browne, M., Donaldson, P., Rose, J., & Rockloff, M. (2015). Understanding gambling related harm: A proposed definition, conceptual framework, and taxonomy of harms. *BMC Public Health,**16*(1), 80. 10.1186/s12889-016-2747-0 10.1186/s12889-016-2747-0 PMC4728872 26818137

[CR24] León-Jariego, J. C., Parrado-González, A., & Ojea-Rodríguez, F. J. (2020). Behavioral intention to gamble among adolescents: Differences between gamblers and non-gamblers—prevention recommendations. *Journal of Gambling Studies,**36*(2), 555–572. 10.1007/s10899-019-09904-6 31673929 10.1007/s10899-019-09904-6

[CR25] Lind, K., Castrén, S., Hagfors, H., & Salonen, A. H. (2022). Harm as reported by affected others: A population-based cross-sectional Finnish Gambling 2019 study. *Addictive Behaviors,**129*, 107263. 10.1016/j.addbeh.2022.107263 35134630 10.1016/j.addbeh.2022.107263

[CR26] Lindeman, S. K., & Selseng, L. B. (2025). Commonalities and Variations. In G. Bischof, R. Velleman, J. Orford, A. Nadkarni, & M. Tiburcio (Eds.),

[CR27] Martin, R. J., Usdan, S., Nelson, S., Umstattd, M. R., LaPlante, D., Perko, M., & Shaffer, H. (2010). Using the theory of planned behavior to predict gambling behavior. *Psychology of Addictive Behaviors,**24*(1), 89–97. 10.1037/a0018452 20307115 10.1037/a0018452

[CR28] Matilainen, R. (2016). Cultural and social meanings of gambling in Finland and Sweden: A historical perspective. In *Random Riches: Gambling Past & Present: Vol. Routledge 2016, first edition* (pp. 119–131). Routledge. 10.4324/9781315603599

[CR29] Matilainen, R., & Raento, P. (2014). Learning to gamble in changing sociocultural contexts: Experiences of Finnish casual gamblers. *International Gambling Studies,**14*(3), 432–446. 10.1080/14459795.2014.923484

[CR30] Meisel, M. K., & Goodie, A. S. (2014). Descriptive and injunctive social norms’ interactive role in gambling behavior. *Psychology of Addictive Behaviors,**28*(2), 592–598. 10.1037/a0036444 24955677 10.1037/a0036444

[CR31] Merkouris, S. S., Rodda, S. N., & Dowling, N. A. (2022). Affected other interventions: A systematic review and meta-analysis across addictions. *Addiction,**117*(9), 2393–2414. 10.1111/add.15825 35129234 10.1111/add.15825 PMC9543616

[CR32] Newall, P., Rawat, V., Hing, N., Browne, M., Tulloch, C., Russell, A. M. T., Li, E., Rockloff, M., & Dellosa, G. (2024). Gambling-related harm to affected others: Lived experience differs by relationship type, gambling severity, life circumstances, and relationship factors. *International Journal of Mental Health and Addiction*. 10.1007/s11469-024-01417-7

[CR33] Oksanen, A., Mantere, E., Vuorinen, I., & Savolainen, I. (2022). Gambling and online trading: Emerging risks of real-time stock and cryptocurrency trading platforms. *Public Health,**205*, 72–78. 10.1016/j.puhe.2022.01.027 35247862 10.1016/j.puhe.2022.01.027

[CR34] Parrado-González, A., Fernández-Calderón, F., Newall, P. W. S., & León-Jariego, J. C. (2023). Peer and parental social norms as determinants of gambling initiation: A prospective study. *Journal of Adolescent Health,**73*(2), 296–301. 10.1016/j.jadohealth.2023.02.033 10.1016/j.jadohealth.2023.02.033 37061904

[CR35] Pisarska, A., & Ostaszewski, K. (2020). Factors associated with youth gambling: Longitudinal study among high school students. *Public Health,**184*, 33–40. 10.1016/j.puhe.2020.03.017 32620298 10.1016/j.puhe.2020.03.017

[CR36] Plant, M. (n.d.). *Drinking careers: Occupations, drinking habits and drinking problems* (Vol. 1979). Tavistock Publications.

[CR37] Reith, G., & Dobbie, F. (2011). Beginning gambling: The role of social networks and environment. *Addiction Research & Theory,**19*(6), 483–493. 10.3109/16066359.2011.558955

[CR38] Riley, B. J., Harvey, P., Crisp, B. R., Battersby, M., & Lawn, S. (2021). Gambling-related harm as reported by concerned significant others: A systematic review and meta-synthesis of empirical studies. *Journal of Family Studies,**27*(1), 112–130. 10.1080/13229400.2018.1513856

[CR39] Rodda, S. N., Dowling, N. A., Thomas, A. C., Bagot, K. L., & Lubman, D. I. (2020). Treatment for family members of people experiencing gambling problems: Family members want both gambler-focused and family-focused options. *International Journal of Mental Health and Addiction,**18*(5), 1318–1334. 10.1007/s11469-019-00143-9

[CR40] Salonen, A. H., Castrén, S., Alho, H., & Lahti, T. (2014). Concerned significant others of people with gambling problems in Finland: A cross-sectional population study. *BMC Public Health,**14*(1), 398. 10.1186/1471-2458-14-398 24758313 10.1186/1471-2458-14-398 PMC4058717

[CR41] Savolainen, I., Sirola, A., Kaakinen, M., & Oksanen, A. (2019). Peer group identification as determinant of youth behavior and the role of perceived social support in problem gambling. *Journal of Gambling Studies,**35*(1), 15–30. 10.1007/s10899-018-9813-8 30465150 10.1007/s10899-018-9813-8 PMC6474853

[CR42] Schunck, R. (2013). Within and between estimates in random-effects models: Advantages and drawbacks of correlated random effects and hybrid models. *The Stata Journal: Promoting Communications on Statistics and Stata,**13*(1), 65–76. 10.1177/1536867X1301300105

[CR43] Schunck, R., & Perales, F. (2017). Within- and between-cluster effects in generalized linear mixed models: A discussion of approaches and the Xthybrid command. *The Stata Journal: Promoting Communications on Statistics and Stata,**17*(1), 89–115. 10.1177/1536867X1701700106

[CR44] Smith, J., Wright, S., Dighton, G., Dymond, S., & Torrance, J. (2025). The influence of family on gambling behaviours: A rapid review of emergent literature. *International Journal of Mental Health and Addiction*. 10.1007/s11469-025-01505-240703248

[CR45] Suomi, A., Lucas, N., Dowling, N., & Delfabbro, P. (2022). Parental problem gambling and child wellbeing: Systematic review and synthesis of evidence. *Addictive Behaviors,**126*, 107205. 10.1016/j.addbeh.2021.107205 34890890 10.1016/j.addbeh.2021.107205

[CR46] Tremblay, J., Savard, A.-C., Blanchette-Martin, N., Dufour, M., Bertrand, K., Ferland, F., Côté, M., & Saint-Jacques, M. (2015). Integrative couple treatment for pathological gambling / ICT-PG: Description of the therapeutic process. *The Canadian Journal of Addiction,**6*(2), 54–61. 10.1097/02024458-201509000-00008

[CR47] Tulloch, C., Browne, M., Hing, N., Rockloff, M., & Hilbrecht, M. (2022). How gambling harms the wellbeing of family and others: A review. *International Gambling Studies,**22*(3), 522–540. 10.1080/14459795.2021.2002384

[CR48] Vassallo, M., DeGiovanni, K., & Montgomery, P. (2023). The efficacy of psychosocial interventions in minimising the harm caused to affected others of problem gambling: A systematic review and meta-analysis. *Journal of Gambling Studies,**39*(4), 1927–1958. 10.1007/s10899-023-10220-3 37294395 10.1007/s10899-023-10220-3 PMC10627969

[CR49] Velleman, R. (1992a). Intergenerational effects—A review of environmentally oriented studies concerning the relationship between parental alcohol problems and family disharmony in the genesis of alcohol and other problems. I: The intergenerational effects of alcohol problems. *International Journal of the Addictions,**27*(3), 253–280. 10.3109/10826089209068741 1563885 10.3109/10826089209068741

[CR50] Velleman, R. (1992b). Intergenerational effects-A review of environmentally oriented studies concerning the relationship between parental alcohol problems and family disharmony in the genesis of alcohol and other problems. II: The intergenerational effects of family disharmony. *International Journal of the Addictions,**27*(4), 367–389. 10.3109/10826089209068748 1563891 10.3109/10826089209068748

[CR51] Velleman, R., Nadkarni, A., Bischof, G., Tiburcio, M., & Orford, J. (2025). First person “Lived Experience” accounts of being an affected family member (AFM). In G. Bischof, R. Velleman, J. Orford, A. Nadkarni, & M. Tiburcio (Eds.),

[CR52] Vepsäläinen, J., Kaakinen, M., Savolainen, I., Hagfors, H., Vuorinen, I., & Oksanen, A. (2024). Online communities as a risk factor for gambling and gaming problems: A five-wave longitudinal study. *Computers in Human Behavior,**157*, 108246. 10.1016/j.chb.2024.108246

[CR53] Vitaro, F., Wanner, B., Brendgen, M., & Tremblay, R. E. (2008). Offspring of parents with gambling problems: Adjustment problems and explanatory mechanisms. *Journal of Gambling Studies,**24*(4), 535–553. 10.1007/s10899-008-9096-6 18498043 10.1007/s10899-008-9096-6

[CR54] Wilson, C., Butler, N., & Quigg, Z. (2024). Harms from other people’s gambling: Associations with an individual’s own gambling behaviours, health risk behaviours, financial problems, general health, and mental wellbeing. *Journal of Gambling Studies,**40*(3), 1–15. 10.1007/s10899-024-10291-w 38489134 10.1007/s10899-024-10291-w PMC11390759

[CR55] Winters, K. C., Stinchfield, R. D., Botzet, A., & Anderson, N. (2002). A prospective study of youth gambling behaviors. *Psychology of Addictive Behaviors,**16*(1), 3–9. 10.1037/0893-164X.16.1.3 11934084 10.1037//0893-164x.16.1.3

[CR56] Wulfert, E., Hartley, J., Lee, M., Wang, N., Franco, C., & Sodano, R. (2005). Gambling screens: Does shortening the time frame affect their psychometric properties? *Journal of Gambling Studies,**21*(4), 521–536. 10.1007/s10899-005-5561-7 16311880 10.1007/s10899-005-5561-7

[CR57] Zhai, Z. W., Yip, S. W., Steinberg, M. A., Wampler, J., Hoff, R. A., Krishnan-Sarin, S., & Potenza, M. N. (2017). Relationships between perceived family gambling and peer gambling and adolescent problem gambling and binge-drinking. *Journal of Gambling Studies,**33*(4), 1169–1185. 10.1007/s10899-017-9670-x 28101835 10.1007/s10899-017-9670-x PMC5515696

